# Oxidative stress and abnormal lipid profile are common factors in students with eating distress

**DOI:** 10.1186/s40337-015-0081-z

**Published:** 2015-11-24

**Authors:** N. Nivedita, G. Sreenivasa, S. Suttur Malini

**Affiliations:** Department of Studies in Zoology, University of Mysore, Mysore, Karnataka India

**Keywords:** Eating disorders, Oxidative stress, Total antioxidant capacity, Lipid profile

## Abstract

Numerous studies on complications associated with eating disorders have been conducted worldwide. However such studies are limited in the Indian scenario. Hence, we attempted to analyse the presence of oxidative stress along with total lipid profiling of students with eating distress in Mysore, South India. A biochemical test panel was conducted using serum samples of controls and subjects. Results were statistically analyzed using SPSS software version 14. Analysis of variance was used to identify significant differences between study groups. Variations in all parameters confirmed the occurrence of oxidative stress and abnormal lipid contents in students prone to eating disorders.

## Background

Unconventional, unbalanced and improper eating practices as a result of severe fixation on one’s physique represent eating disorders. Their onset revolves around the exposure of an adolescent mind to overpublicized fitness body images on different platforms of mass media [1]. The Academy of Eating Disorders (AED) emphasizes that eating disorders should be dealt with equal gravity as that of bipolar disorder, schizophrenia and depression as they all belong to the same category i.e., Biologically Based Mental Illness (BBMI) [2].

Oxidative stress is the condition caused when the body’s proficiency to incapacitate Reactive Oxygen Species (ROS) is diminished [3]. Enzymes like superoxide dismutase (SOD), catalase (CAT) and glutathione peroxidase form the body’s defence system to neutralise ROS [3]. The estimation of lipid peroxidation (LPO) by-products and these enzymes aid in identifying the extent of oxidative stress existing in an individual. In addition to this, nitrosative stress also plays an important role in the advancement of these disorders.

Earlier reports have revealed the presence of both oxidative stress and nitrosative stress among patients with Anorexia Nervosa (AN). The manifestation of lipid peroxidation products [4], conflicting SOD levels [5, 6], and discrepancies in catalase activity [6] have been reported previously. Similarly, reduction in nitric oxide synthase (NOS) scavenging activity [5] has also been reported. Parallel studies of participants with Bulimia Nervosa (BN) and Binge Eating Disorder (BED) have not been found.

Premature deaths in AN patients due to cardiovascular diseases have been widely explored in research articles. Variations in all the parameters of total lipid profiles have been reported earlier. High total cholesterol (TC), triglycerides (TG), high density lipoprotein (HDL) but normal low density lipoprotein (LDL) [7] were the outcomes of one study whereas another study depicted similar results with an increase in LDL [8]. High dietary consumption of fats and lipids in individuals with BN and BED may give rise to elevated values in the total lipid profiles of such patients. Increased serum cholesterol have been observed in patients with BN [9] however, such studies were not available for individuals with BED.

Studies involving oxidative and nitrosative stress and total lipid profiling of patients with eating disorders have not been conducted in India. To fill this lacuna we conducted the current study with a non-clinical sample of students with eating distress. The study was conducted using the student population of the city of Mysore, South India. To the best of our knowledge, this study is the first of its kind to be conducted in India.

## Methodology

A preliminary survey was conducted using EAT-26 (Eating Attitudes Test - 26) [10] and BES (Binge Eating Scale) [11] questionnaires spanning 1600 students across the city of Mysore, South India; aged 15–25 years. Among the 417 students who scored high in either of the questionnaires, 35 students (18 EAT-26 high scorers and 17 BES high scorers) were recruited as participants. Along with their scores, altered BMI and abnormal eating practices were prerequisites for inclusion. Similarly 35 students with very low scores in both the questionnaires and normal BMI participated as controls.

On obtaining ethical clearance from the Institutional Human Ethical Clearance Committee of University of Mysore (IHEC-UOM No.05/M.Sc/2013-14), blood samples were collected from both participants following an overnight fast. Serum separated from these samples was subjected to various estimations namely SOD, Catalase, Lipid Peroxidation and NOS scavenging activity. Total lipid profile was determined using diagnostic kits from Erba Mannheim group, manufactured by Transasia Bio- Medicals Ltd., India and by using the Friedewald equation.

Results were analysed using SPSS software version 14.01. Mean and standard error were used for comparisons. Analysis of variance (ANOVA) was employed to distinguish any significant difference existing between the different groups and *p* < 0.05 was considered to be significant.

## Results

The mean scores obtained by the controls in the EAT-26 and BES questionnaires were 6.22 ± 0.56 and 7.51 ± 0.60 respectively. The high scorers of EAT-26 displayed a mean score of 38.33 ± 1.81 in the 26 item Eating Attitudes Test. Similarly, a mean score of 32.64 ± 0.99 was observed in the high scorers of BES in the Binge Eating Scale. The mean ages of the study groups were found to be similar i.e., 22.34 ± 0.22 years, 22.44 ± 0.24 years and 21.58 ± 0.42 years for controls, EAT-26 high scorers and BES high scorers. The mean BMI of the study groups were 20.88 ± 0.57 kg/m^2^ for controls, 18.69 ± 0.87 kg/m^2^ for EAT-26 high scorers and 23.62 ± 0.97 kg/m^2^ for BES high scorers. Female participants of the study groups displayed expected variations in the mean BMI values; female high scorers of EAT-26 presented a low mean BMI of 17.68 ± 1.10 kg/m^2^ categorised as underweight, female controls exhibited mean BMI within normal range (21.19 ± 0.67 kg/m^2^) and finally the female high scorers of EAT-26 displayed a mean BMI of 25.28 ± 1.34 kg/m^2^ categorised as overweight. However, the mean BMI displayed by the male participants of the study groups did not seem to differ significantly with the values being 20.42 ± 1.03 kg/m^2^, 20.71 ± 1.13 kg/m^2^ and 21.25 ± 0.84 kg/m^2^ for male controls, EAT-26 high scorers and BES high scorers respectively.

The estimation of catalase activity & SOD found that both the subject groups exhibited reduced catalase activity but an augmented SOD activity when compared to the controls. Malondialdehyde (MDA), a by-product of lipid peroxidation was found to be present in all the study groups. The difference between the groups in this case was statistically significant (*p* < 0.05). NOS scavenging activity was higher in the participants than in controls (Table 1).

**Table 1:** Comparison of mean values of superoxide dismutase (SOD), lipid peroxidation (LPO), catalase (CAT) and nitric oxide synthase (NOS) scavenging activity

Biomarkers	Type	Number	Mean ± SE	df	F	*p*
SOD (Units of SOD/mg of protein)	Control	35	0.0440 ± 0.005	2	2.118	0.128
	EAT-26	18	0.0668 ± 0.01			
	BES	17	0.0646 ± 0.01			
LPO (n moles of MDA/mg of protein)	Control	35	0.1098 ± 0.01	2	9.357	0.000*
	EAT-26	18	0.2894 ± 0.05			
	BES	17	0.3026 ± 0.05			
CAT (k/ml)	Control	35	0.0370 ± 0.01	2	0.987	0.378
	EAT-26	18	0.0129 ± 0.004			
	BES	17	0.0182 ± 0.01			
NOS (%)	Control	35	31.24 ± 2.09	2	9.302	0.000*
	EAT-26	18	50.41 ± 4.85			
	BES	17	47.39 ± 5.27			

Comparison of mean values of SOD, LPO, CAT and NOS scavenging activity between controls and high scoring participants of EAT-26 and Binge Eating Scale (BES)

* *p* < 0.05

**Fig. 1:**
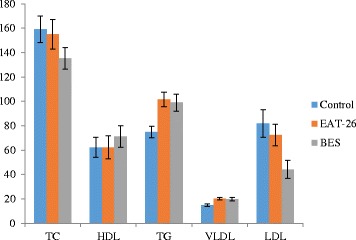
Comparison of mean values of lipid profile between controls and high scoring participants of EAT-26 and BES (TG & VLDL displayed *p* < 0.05 indicating significant value)

Total lipid profile parameters varied erratically in all the groups (Fig. 1). Mean TG and VLDL values were statistically significant in the different groups. No distinct correlation between BMI and lipid profile of the study groups was detected.

## Inferences

The causes and effects of eating disorders need to be assessed through specific biomarkers in order to obtain an advanced perception of these disorders. While such studies are common worldwide, in the Indian population, they are rare.

In our study we observed increased SOD activity and decreased catalase activity in participants with EAT-26 high scores. Similar results were observed in another study with AN patients [6]. Results from another analysis expressed the presence of LPO by-products in AN patients [4] which paralleled with our study where elevated MDA was observed in the high scorers of EAT-26. From the above results we conclude that oxidative stress occurs in people with high EAT-26 scores. A persistent diminution in food consumption generates more ROS in AN due to damage of enzymatic and non-enzymatic oxidative systems [12]. This statement from an earlier report explains our findings.

A previous study confirmed an upsurge in NO production and NOS iso-forms [13]. Likewise, we noticed elevated NOS scavenging activity in high scorers of EAT-26. A similar outcome in the high scorers of BES was also observed. However, no accounts of associations between BED and oxidative and nitrosative stress have been made as yet. Additional in-depth analysis of BED needs to be carried out.

Anorexia Nervosa patients suffer from cardiovascular diseases is a well acknowledged fact. Consequently numerous investigations on the lipid profile of AN patients have been undertaken. Hypercholesterolemia was noticed in a substantial portion of EAT-26 high scorers, even though the participants from our study did not demonstrate higher TC. This is consistent with other studies where AN patients frequently presented with hypercholesterolemia [14, 15]. In accordance with earlier studies [7, 8] we found increments in various parameters of lipid profile among the at- risk groups. Elevated resorption of exogenic cholesterols could be one of the reasons for hypercholesterolemia in AN patients [16]. This may possibly explain the hypercholesterolemic condition noticed in EAT-26 high scorers. In BES high scorers, the variations in lipid profile are clearly accredited to the unstable and unbalanced eating practices of binge eaters including high intake of fat rich nutrition.

Eating disorders are now commonplace in India, however their diagnosis in the general population of the country is extremely rare. Multifaceted researches involving oxidative and nitrosative stress and total lipid profiling of patients with these disorders and their effects are needed. Our study was limited owing to the absence of a suitable diagnostic tool to clinically validate the manifestation of eating disorders in our subjects. However, this preliminary report may be first in India, assessing the occurrence of oxidative stress, nitrosative stress and variations in the total lipid profile of a non-clinical sample of students with eating distress.

## Abbreviations

AED: academy of eating disorders
AN: anorexia nervosa
ANOVA: analysis of variance
BBMI: biologically based mental illness
BED: binge eating disorder
BES: binge eating scale
BMI: body mass index
BN: bulimia nervosa
CAT: catalase
EAT-26: eating attitudes test-26
HDL: high density lipoprotein cholesterol
LDL: low density lipoprotein cholesterol
LPO: lipid peroxidation
MDA: malondialdehyde
NOS: nitric oxide synthase
ROS: reactive oxygen species
SOD: superoxide dismutase
TC: total cholesterol
TG: triglycerides
VLDL: very low density lipoprotein cholesterol

## References

[1] Moriarty CM, Harrison K (2008). Television exposure and disordered eating among children: a longitudinal panel study. J Commun.

[2] Klump KL, Bulik CM, Kaye WH, Treasure J, Tyson E (2009). Academy for eating disorders position paper: eating disorders are serious mental illnesses. Int J Eat Disord.

[3] Finkel T, Holbrook NJ (2000). Oxidants, oxidative stress and the biology of ageing. Nature.

[4] Tajiri K, Shimizu Y, Tsuneyama K, Sugiyama T (2006). A case report of oxidative stress in a patient with anorexia nervosa. Int J Eat Disord.

[5] Pereira NR, Moss MB, Assumpção CR, Cardoso CB, Mann GE, Brunini TM (2010). Oxidative stress, L-arginine–nitric oxide and arginase pathways in platelets from adolescents with anorexia nervosa. BCMD.

[6] Moyano D, Sierra C, Brandi N, Artuch R, Mira A, García‐Tornel S (1999). Antioxidant status in anorexia nervosa. Int J Eat Disord.

[7] Žák A, Vecka M, Tvrzicka E, Hrubý M, Novak F, Papežová H (2005). Composition of plasma fatty acids and non-cholesterol sterols in anorexia nervosa. Physiol Res.

[8] Ohwada R, Hotta M, Oikawa S, Takano K (2006). Etiology of hypercholesterolemia in patients with anorexia nervosa. Int J Eat Disord.

[9] Pauporte J, Walsh BT (2001). Serum cholesterol in bulimia nervosa. Int J Eat Disord.

[10] Garner DM, Olmsted MP, Bohr Y, Garfinkel PE (1982). The eating attitudes test: psychometric features and clinical correlates. Psychol Med.

[11] Gormally J, Black S, Daston S, Rardin D (1982). The assessment of binge eating severity among obese persons. Addict Behav.

[12] Moser S, Plaetzer K, Radak Z, Thun-Hohenstein L, Krammer B (2008). Caloric restriction and anorexia nervosa with regard to the oxidant-antioxidant balance. Ber nat-med Ver Salzburg.

[13] Vignini A, D’Angelo M, Nanetti L, Camilloni MA, Cester AM, Faloia E (2010). Anorexia nervosa: a role for L‐arginine supplementation in cardiovascular risk factors?. Int J Eat Disord.

[14] Nestel PJ (1974). Cholesterol metabolism in anorexia nervosa and hypercholesterolemia. J Clin Endocrinol Metab.

[15] Mira M, Stewart PM, Vizzard J, Abraham S (1987). Biochemical abnormalities in anorexia nervosa and bulimia. Ann Clin Biochem.

[16] Zák A, Vecka M, Tvrzická E, Novák F, Papezová H, Hrubý M (2002). Lipid metabolism in anorexia nervosa. Cas Lek Cesk.

